# Distribution, Population Biology, and Trophic Ecology of the Deepwater Demersal Fish *Halosauropsis macrochir* (Pisces: Halosauridae) on the Mid-Atlantic Ridge

**DOI:** 10.1371/journal.pone.0031493

**Published:** 2012-02-22

**Authors:** Odd Aksel Bergstad, Laura Clark, Hege Øverbø Hansen, Nicola Cousins

**Affiliations:** 1 Institute of Marine Research, Flødevigen, His, Norway; 2 Scottish Natural Heritage, Inverness, Scotland, United Kingdom; 3 University of Aberdeen, Aberdeen, Scotland, United Kingdom; Ecole Normale Supérieure de Lyon, France

## Abstract

*Halosauropsis macrochir* ranked amongst the most abundant and widespread demersal fishes on the mid-Atlantic Ridge of the North Atlantic (Iceland-Azores) with greatest abundance at 1700–3500 m. All sizes, ranging from 10–76 cm total length, occurred in the area without any apparent spatial pattern or depth trend. Using otolith sections displaying growth increments assumed to represent annuli, the age range recorded was 2–36 years, but most individuals were <20 years. Length and weight at age data were used to fit growth models. No differences between sexes in length and weight at age were observed. The majority of samples had a surplus of males. Diet analysis showed that *H. macrochir* feeds on Crustacea, Teleostei, Polychaeta, and Cephalopoda, but few prey could be identified to lower taxonomical levels. The mid-Atlantic Ridge constitutes a major portion of the North Atlantic living space of the abyssal halosaur where it completes its full life cycle, primarily as an actively foraging euryophagous micronekton/epibenthos and infauna feeder, becoming a partial piscivore with increasing size.

## Introduction

This study was an element of a comprehensive investigation of the animals associated with the mid-Atlantic Ridge of the North Atlantic [1], including also analyses of life history diversity and autecology of selected species. Amongst demersal fishes, *Halosauropsis macrochir* (Günther 1878), the abyssal halosaur, was selected. The species belongs to the spiny eel family Halosauridae of the order Notacanthiformes. It has a tapered caudal region and grows to about 90 cm in length (e.g. [2], [3]). Benthic juveniles and adults are widespread and common, found at 1100–3300 m depth, in the Eastern Atlantic (Ireland to Mauritania and South Africa), Western Atlantic (Canada to 25°N, and southern Brazil), Indian Ocean and Western Pacific (Australia, New Zealand, Japan) [2]–[5].

*Halosauropsis macrochir* is also an abundant benthic fish on the Mid-Atlantic Ridge [6], [7]. Information on its life history characteristics is very sparse but scattered data on trophic ecology suggests a benthic lifestyle with gut contents comprising infaunal and epifaunal organisms such as crustaceans, polychaetes, echinoderms, and small fish, as well as detritus and sediments [4], [8]. From diet accounts associated with parasitology studies the species is thought to be an important apex predator with a largely crustacean diet consisting of Isopoda, Amphipoda and Copepoda [9]–[11]. Submersible observations showed the species hovering near the seabed or resting on the bottom [7], [8].

We enhance the knowledge of distribution, age, growth, and feeding ecology of *H. macrochir* from its major mid-ocean habitat in the North Atlantic. A prerequisite for some of the studies was developing methodology for age determination of individual fish using otolith growth zones assumed to represent annuli.

## Materials and Methods

### Sampling

**Figure 1:**
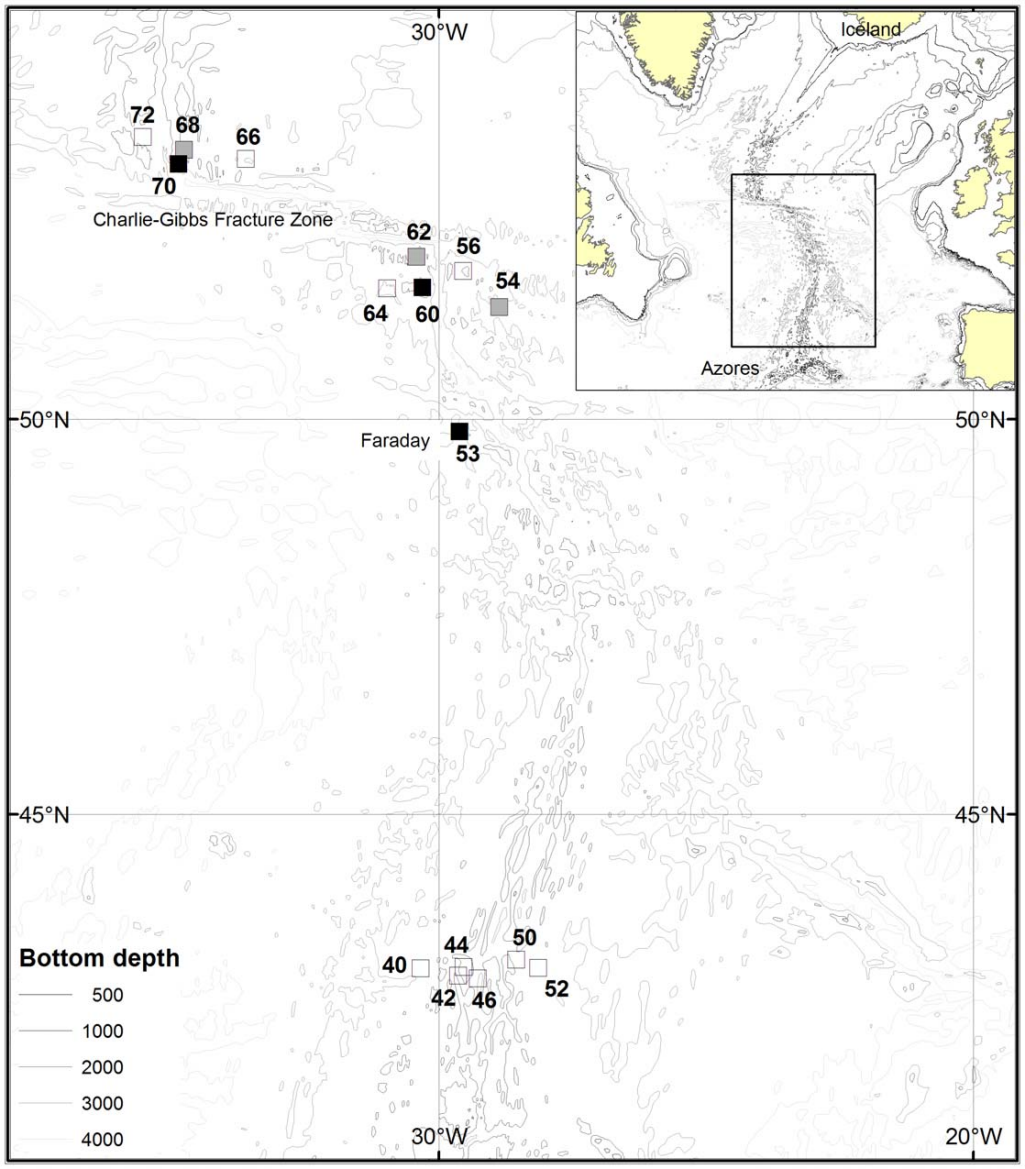
Study area and locations sampled with the bottom trawl. RV G.O.Sars, July 2004.

At the mid-Atlantic Ridge locations shown in Fig. 1, spanning the depth range 800–3532 m, bottom trawling was carried out as elements of the July 2004 MAR-ECO expedition on the RV *G.O.Sars* [12]. Of the 22 bottom trawl tows conducted, 17 were considered valid based on an assessment of the technical quality of the operation. The bottom trawl deployed was a Campelen 1800 shrimp trawl [13]. Horizontal opening between the upper bridles at the wing tips was 17 m and vertical opening was 4.5 m, and a rockhopper ground gear was used. Further details on gear and operations, and other data sampled in the trawl locations, were provided by [6] and [12].

The primary target for the bottom trawling was demersal fish (benthic and benthopelagic species). All fish were identified to the lowest possible taxonomic level, and catches of each species were weighed and counted. From most trawls samples the entire catch or random subsamples were frozen pending confirmation of species identifications, deposition in the collections at the Bergen Museum in Norway, and various follow-on studies. A random sub-sample of 240 *H. macrochir* was selected for further studies of biology and diet. After thawing, specimens were re-weighed (to nearest g) and total length (mm) measured from the snout to the tip of the tail. Stomachs and otoliths were extracted and conserved in 70% ethanol.

### Age determination and growth analyses

The largest otolith, the sagitta, was selected for developing age determination techniques. For most individuals both left and right sagittae were available. The greatest length of the otoliths along the antero-posterior axis was measured to the nearest 0.01 mm using digital calipers. After removing all remaining tissue under a dissecting microscope, otoliths were dried in a 50°C oven for 2 hours.

Due to their small size, initial attempts were made to reveal growth zones in intact otoliths without any pre-processing. But the otoliths appeared opaque in both transmitted and reflected light, and this method was abandoned, even for small specimens. Attempt were also made to reveal interior growth patterns by grinding otolith surface by hand, using Buehler grinding paper for metallography (grit of P1200) whilst immersed in water. The otolith is roughly hemispherical, and grinding removed material from the flat surface. However, this strategy also proved unsuccessful. A more successful technique was to embed dried otoliths in a clear epoxy resin (2∶1, epoxy∶hardener) to cut thin sections. Transverse sections were cut using a low speed ISOMET saw with double diamond-coated blades. Sections of approximately 0.1 mm thickness were obtained and mounted onto microscope slides using clear epoxy resin. The otoliths are small and brittle and many sections fractured during the process. The sections were examined under a light microscope with 25–100× magnification. In total otoliths from 162 specimens were successfully sectioned and mounted, and 105 displayed growth zones resembling annuli. Otoliths from specimens of TL<30 cm could not be sectioned nor viewed intact, hence no otolith age estimates were obtained for the smallest fish.

Age determination techniques have not been developed previously for *H. macrochir*, hence to determine the most suitable methodology for achieving consistent counts of presumed annuli, a multi-step intercalibration exercise involving several readers was carried out. Initially three readers counted presumed annulli on the same set of otolith sections without access to other readers counts nor fish size (Table 1). In cases where deviations were large (e.g. Label no. 31172, 31256), the sections were discussed amongst the readers with a view to explain the discrepancies. Similarly, sections with no or low deviation between readers were discussed. While this process resulted in agreed interpretation principles and guidelines, precision remained comparatively low. Many sections were not clear due both to weak zonation and inferior technical quality of the sections of the small and brittle otoliths.

**Table 1:** Independent readings of otolith sections for interpretation discussions.

Label No	Total Length (mm)	Total Weight (g)	Reader 1	Reader 2	Reader 3
31802	630	170	21	20	19
34126	690	345	16	16	20
30802	450	54	7	4	5
30592	470	80	11	8	7
30065	610	167	14	11	16
31172	550	115	8	15	5
31256	470	91	5	11	7
33258	570	153	9	9	9
30653	570	107	7	4	6
31887	500	125	6	6	6
33174	560	161	7	8	6
30527	500	99	6,7	6	7
30107	470	104	10	11	10
31536	510	131	8	9	8
33902	510	118	8	10	10
33860	660	326	15	10	14
33468	450	65	5	5	5
31929	590	173	12	15	10

The parameters of the von Bertalanffy growth equation were estimated for males and females by a non-linear least squares procedure using the nls function and the FSA package in R. Confidence bands for the growth curves in terms of length and weight were estimated by bootstrapping. For each bootstrap iteration the parameters of the resulting growth equation were estimated. Predicted length- and weight-at-age were then calculated for the entire set of bootstrap estimated equations and 95% confidence bounds calculated as the 2.5 and 97.5 percentiles. Small unsexed specimens (8 specimens) were assigned at random to either females or males before fitting the growth curves. Individuals of TL<30 cm were not aged using otoliths, but tentatively assumed to belong to Age-group 1.

### Diet analysis

Stomachs were cut open and contents for each specimen placed in 70% ethanol for later analysis. Contents were placed on plankton gauze over a glass dish, partially submerged in 70% ethanol. Prey items were then identified to the lowest possible taxonomic level. Once analysis was completed, excess liquid was extracted with tissue paper and contents sorted into prey categories were weighed to the nearest 0.01 g. In cases where the contents weighed <0.01 g, items were assigned the weight of 0.005 g.

Due to the variable digestion state of the contents and a high proportion of unidentifiable prey, most of the contents could only be assigned to major taxonomic categories. Unidentified remains containing fish scales or bones were assigned as ‘unidentified Teleostei’. Legs and other body parts with an exoskeleton were often allocated to Crustacea and could only in a few cases be assigned to lower taxa. Presence of fine hairs and bristles were taken as indications of Polychaeta. Enumeration of prey items was seldom possible, and diet composition was determined using weights, i.e.:
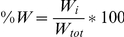



*W_i_* = Weight of prey category *i*

*W_tot_* = Weight of total stomach contents of non-empty stomachs.

Exceptional large single prey items tend to skew and distort weight distributions, hence the *%W* distributions using all prey items were compared to corresponding distributions after first eliminating single items >1 g and then eliminating items weighing >2 g.

## Results

### Distribution and abundance

**Figure 2:**
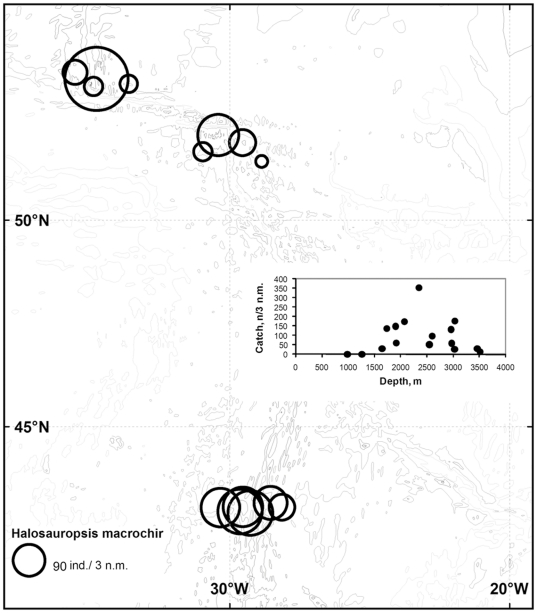
Abundance of *Halosauropsis macrochir* by area and depth. Abundance is expressed as catch in numbers per 3 nautical miles (n.m) towed.

**Figure 3:**
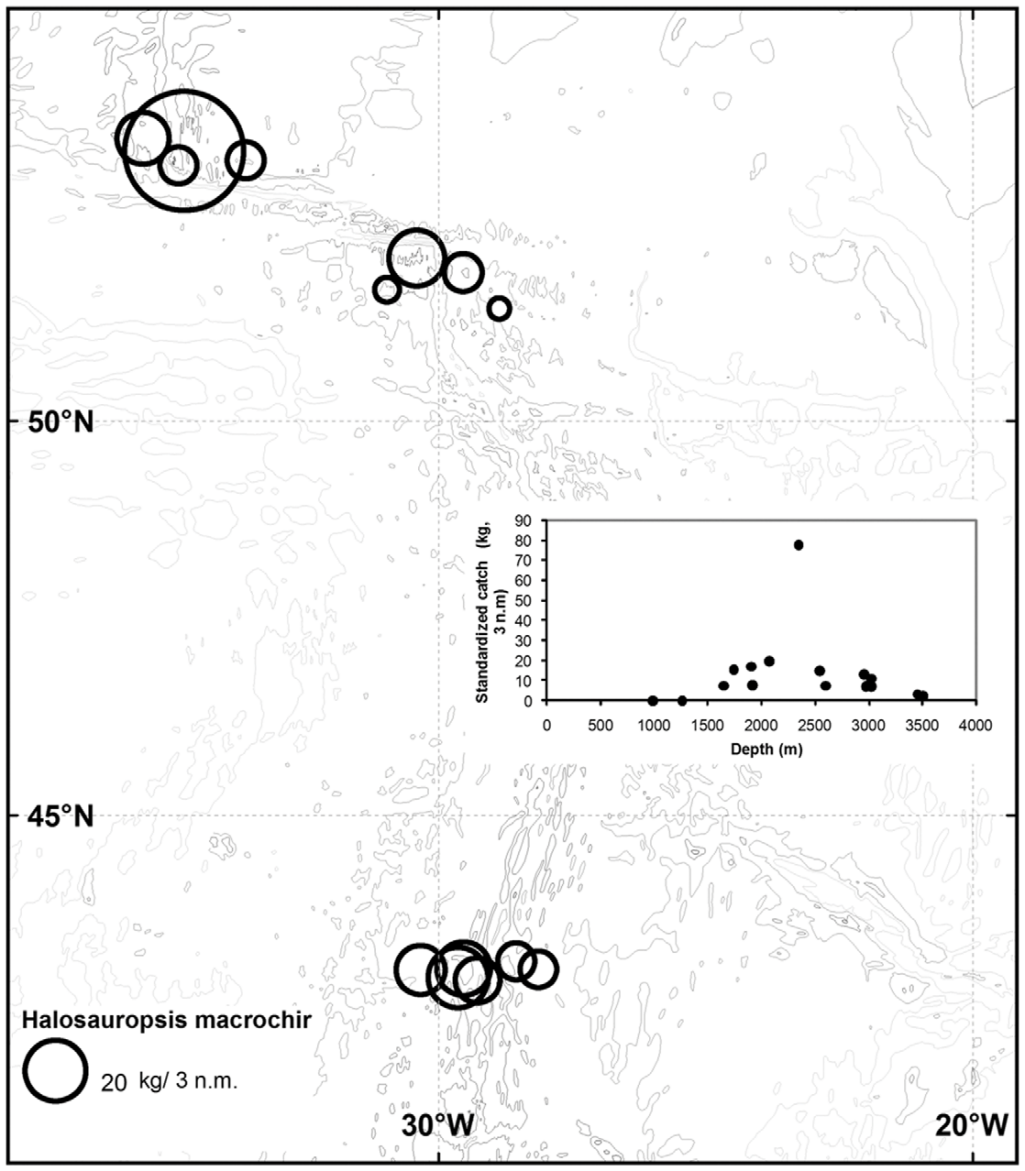
Biomass of *Halosauropsis macrochir* by area and depth. Abundance is expressed as catch in weight (kg) per 3 nautical miles (n.m.) towed.

A total of 640 *H. macrochir* were captured in the 17 successful bottom trawls. Figure 2 and 3 shows the distribution expressed as catch rate in terms of numbers and weight, respectively. The species occurred along the entire ridge section sampled, but was most abundant in the depth range 1700–3500 m, and uncommon or absent on the shallowest slopes and hills.

### Length and sex distributions

**Figure 4:**
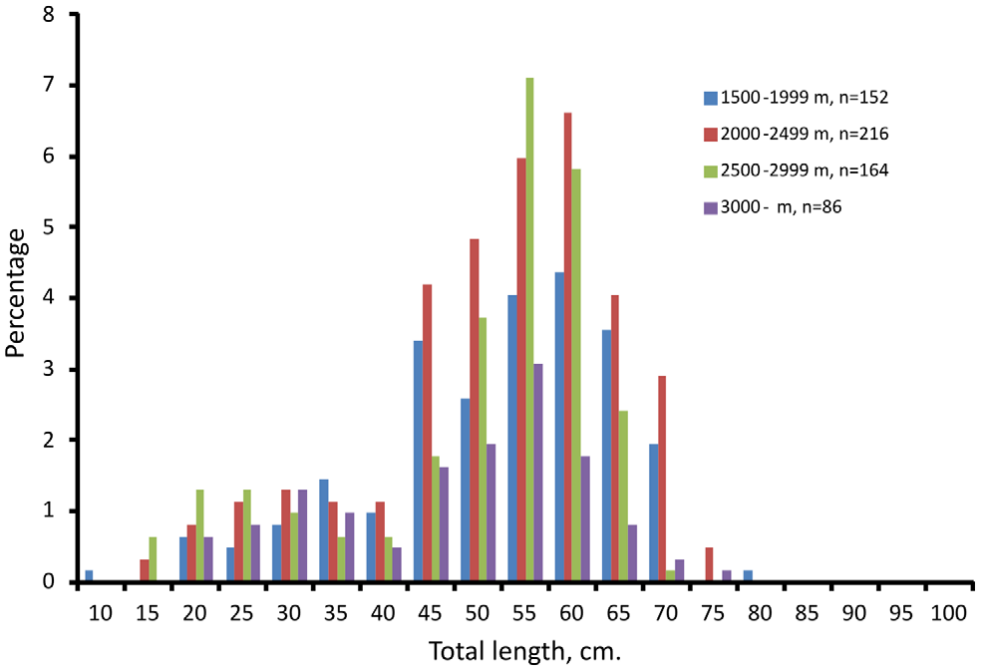
Size frequency distributions of *Halosauropsis macrochir* by depth zone. n = number of specimens measured.

Length frequency distributions showed that individuals from 10 to 76 cm occurred in the area, and at all depths the distributions were skewed towards large fish (Fig. 4). No segregation of sizes by depth was apparent, and female and male distributions were similar.

**Figure 5:**
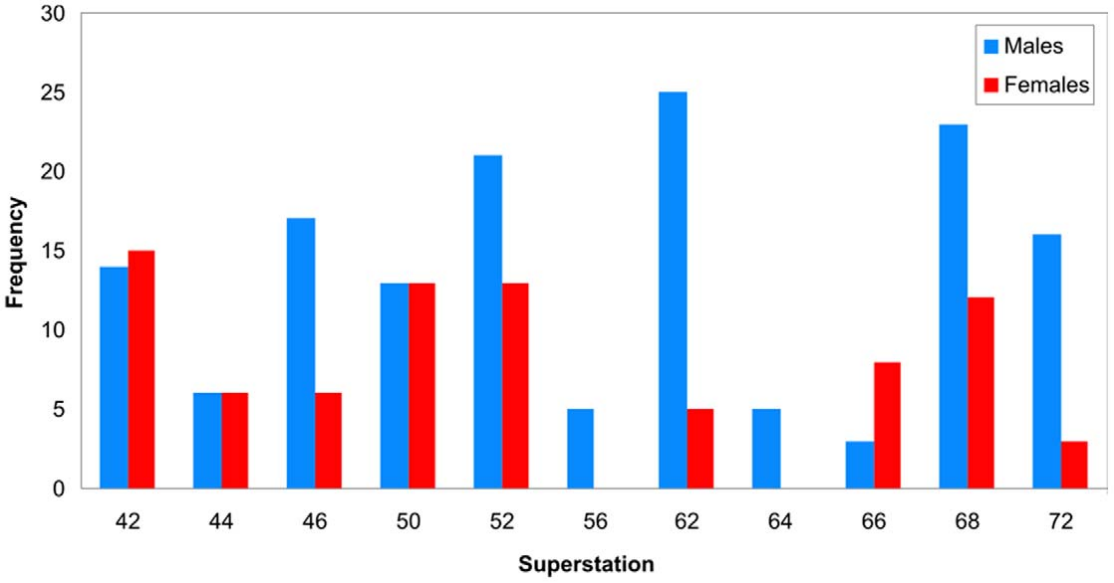
Proportions of males and females at individual locations shown in **Figure 1**.

The dataset showed a predominance of males over females (148 males and 81 females), but fish smaller than 25 cm could generally not be sexed. The uneven sex ratio was observed at 7 out of 11 locations and was statistically significant (Fig. 5, Chi-square test, p = 0.012, d.f. = 10).

### Longevity and Age composition

Independent age readings by three readers (Table 1) indicate the level of variation amongst readers to be expected for this species showing rather unclear growth increments. The average Coefficient of Variation (CV) and index of precision (D) as proposed by [14] were 16.7% and 9.6%, respectively.

**Figure 6:**
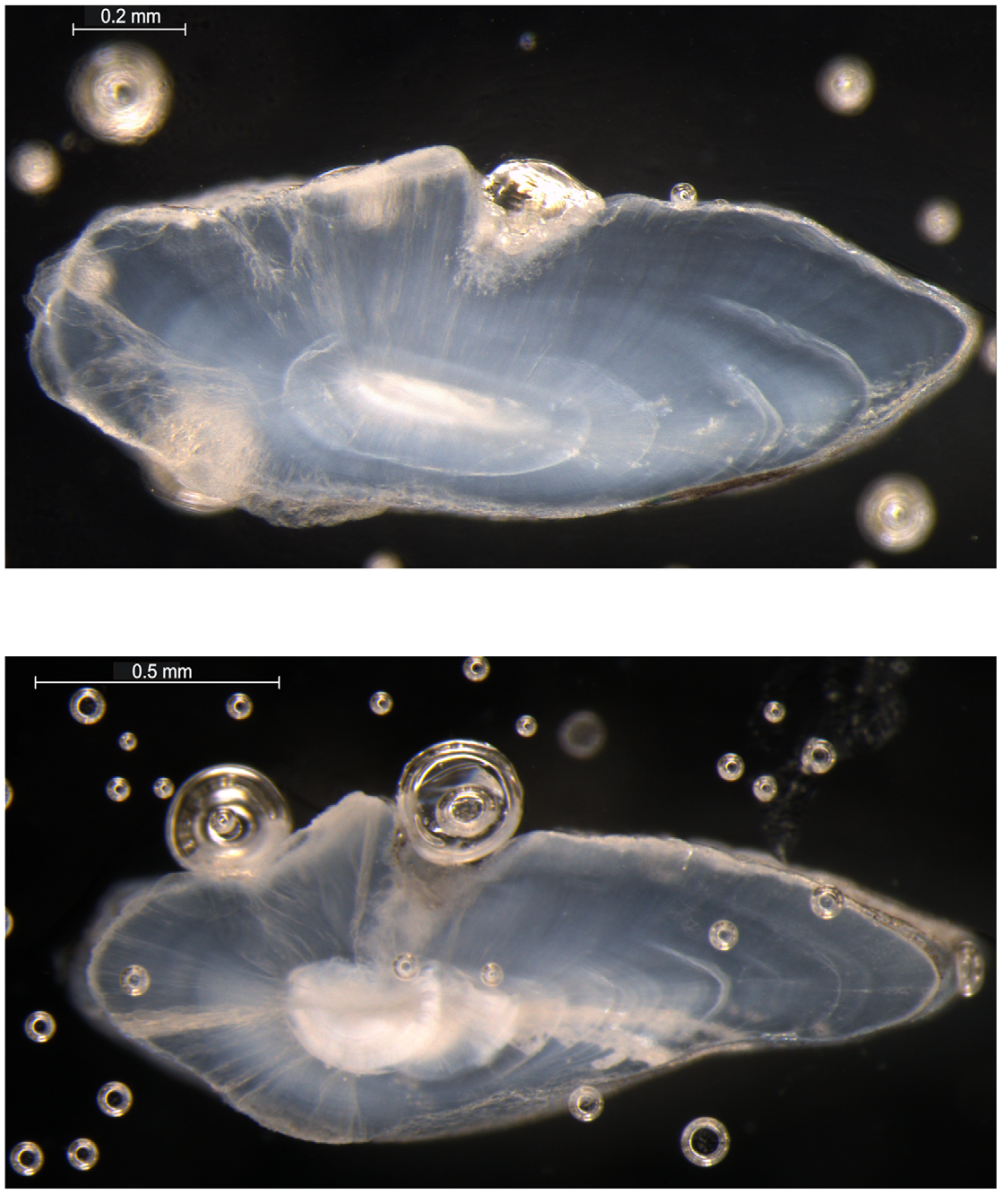
Transverse section of otoliths (sagittae) of *Halosauropsis macrochir*, displaying growth zones assumed to be annuli. Photo: Lise Heggbakken, Institute of Marine Research, Norway.

**Figure 7:**
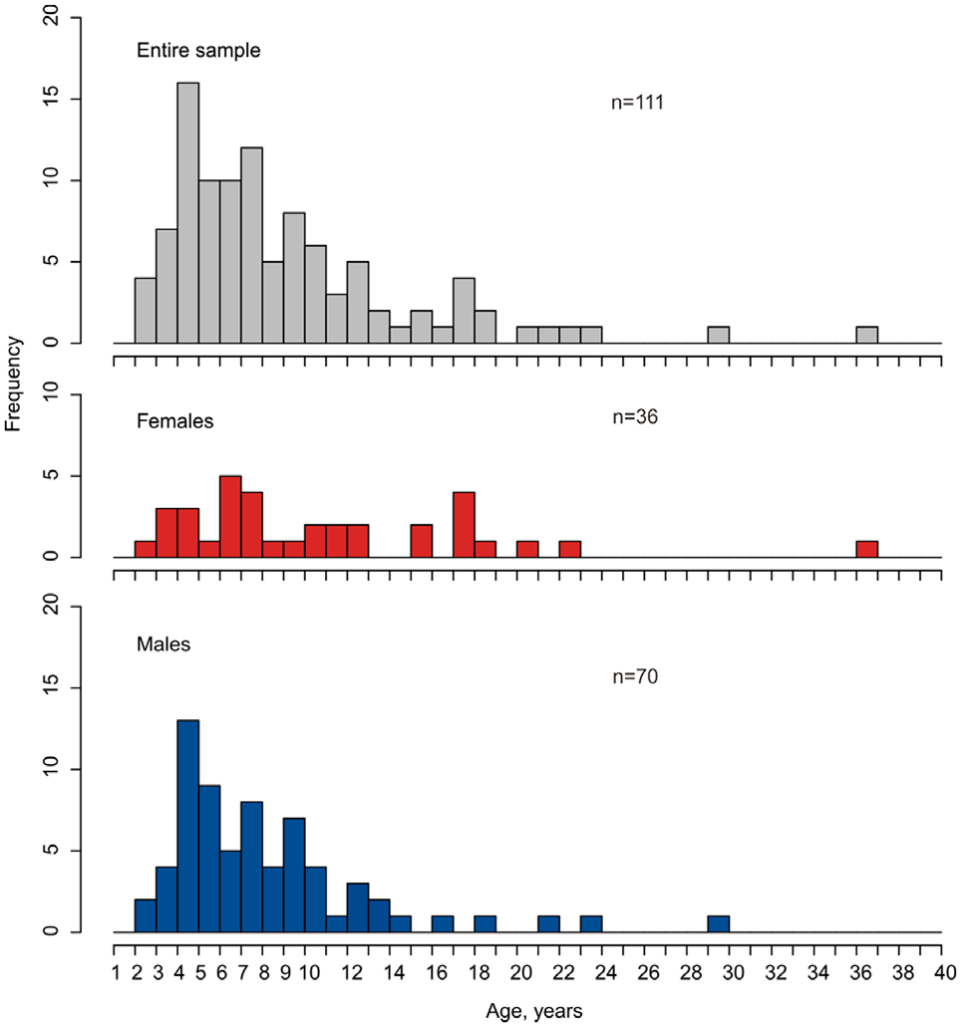
Age composition of *Halosauropsis macrochir*, based on age determined from otolith sections.

There was a linear relationship between otolith size (maximum antero-posterior distance) and total body length. Figure 6 shows examples of otolith sections and growth zones regarded as annuli. Age frequency distributions for both males and females were unimodal and skewed towards rather high ages (Fig. 7). The overall age range was 2–36 years, but only very few fish were older than 20 years.

### Length-weight relationship and growth

**Figure 8:**
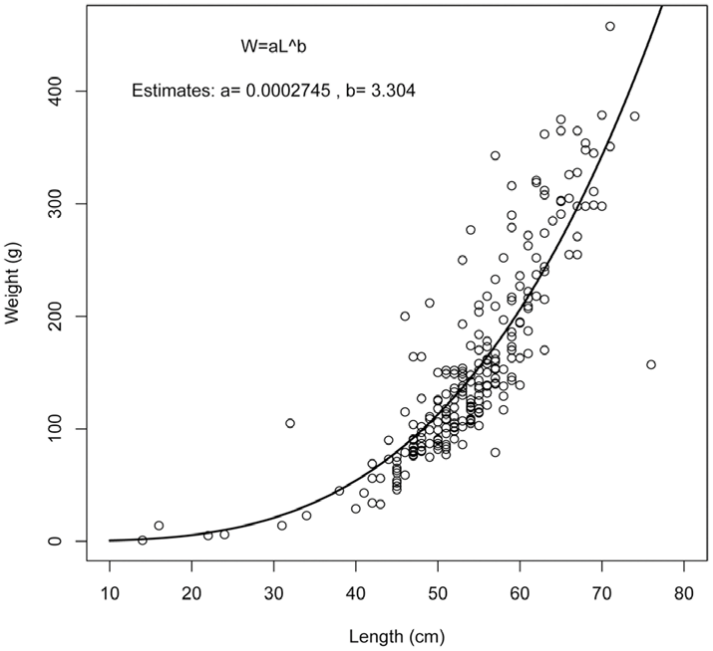
Length-weight relationship of *Halosauropsis macrochir*. Length is total length from snout to tip of caudal fin, and weight is ungutted total body weight.

**Figure 9:**
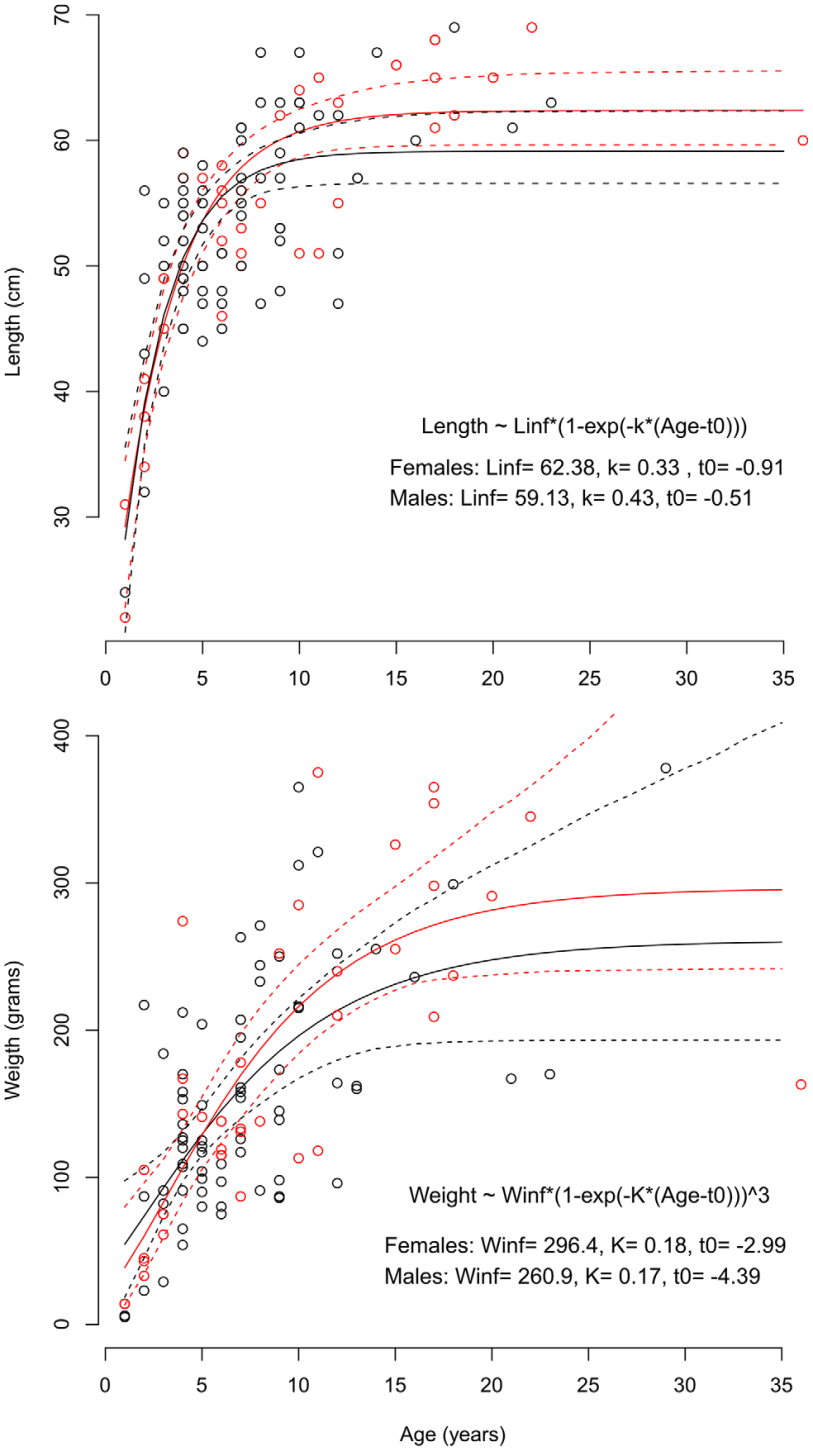
Scatter plots of length-at-age (upper) and weight-at-age (lower) of individual *Halosauropsis macrochir*, and fitted von Bertalanffy growth functions. Red lines and symbols – females. Black lines and symbols - males, Dashed lines are 95% confidence bands for female and male growth curves.

The length-weight relationship of *H. macrochir* is given in Figure 8, and length and weight at age in Figure 9, with fits of the Von Bertalanffy growth functions for males and females. The high variability resulted in rather poor fits. In particular, the intercept with the age axis, t_0_, is estimated with low precision due to the imbalance between young and old fish in the dataset used to fit the models. There was no indication of difference in growth between sexes. Residuals increased somewhat with age, but showed no pattern of deviation from the growth model at particular age ranges.

### Diet

**Figure 10:**
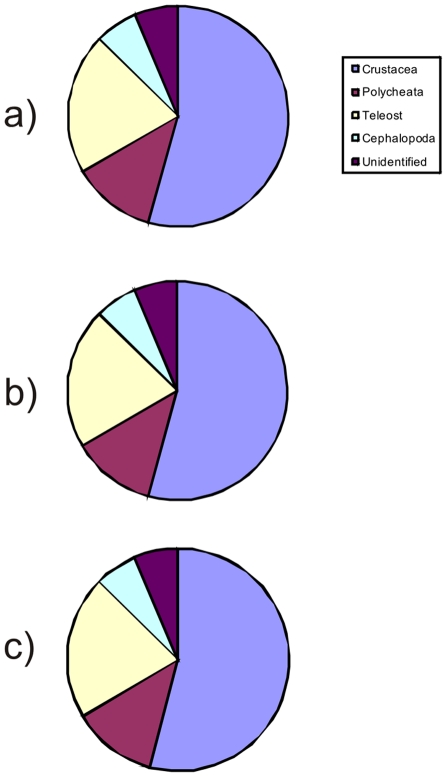
Stomach content composition in terms of weight, *Halosauropsis macrochir*. a) All stomachs and prey items; b) Prey items >1 g removed; c) Prey items >2 g removed.

**Figure 11:**
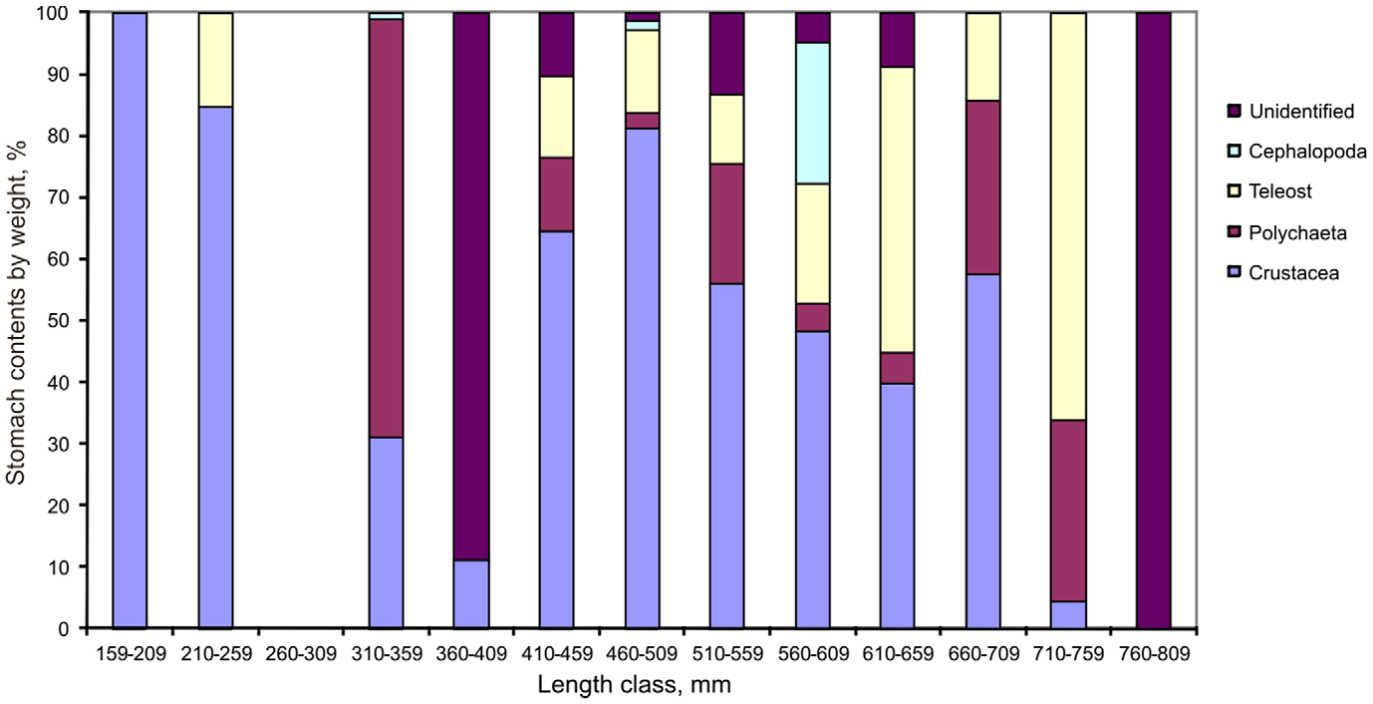
Stomach content composition of different size classes of *Halosauropsis macrochir*.

Of the 240 stomachs dissected 234 had contents, and the majority contained just a single prey taxon (category). Most food items were extensively digested and could only be identified to high taxonomical levels (order, class). About half of the contents were Crustacea, followed by Teleostei, Polychaeta and Cephalopoda (Fig. 10). Several large individual prey items were found, but removal of these items from diet analysis did not change the overall diet composition significantly. Figure 11 suggests that the proportion of teleost prey increased with predator length.

**Figure 12:**
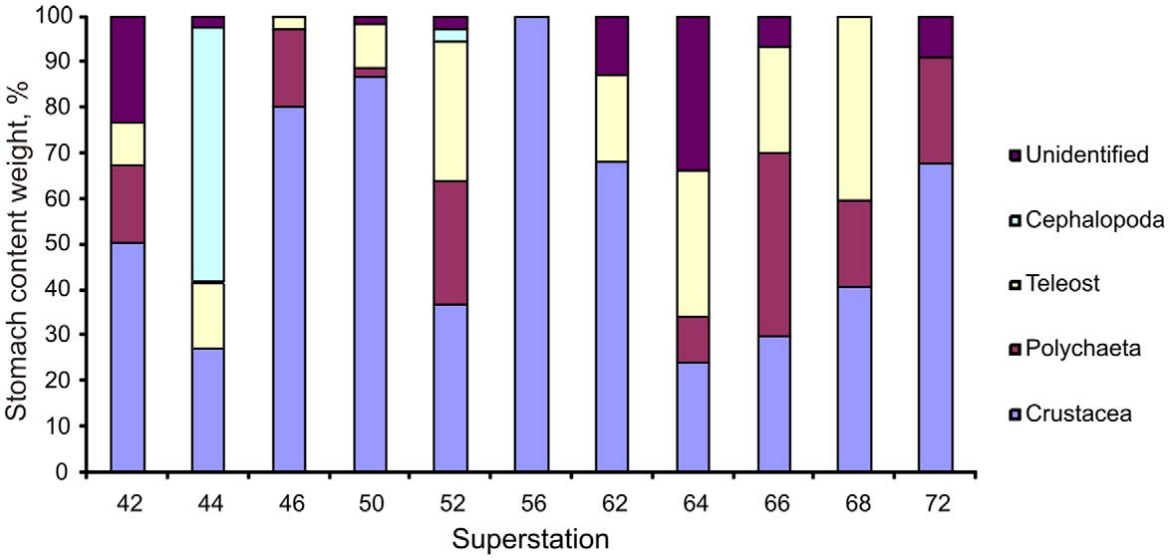
Stomach content compositions (% weight) at different locations (**Fig. 1**).

Cephalopoda were only found in stomachs from SS 44 and 52 (Fig. 12), in the southern sub-area sampled. There was an increased proportion of teleost in the diets of fish in the northern sub-area and a corresponding larger proportion of Crustacea in the south.

The crustacean diet component was separated further into lower taxa. Amphipods, ostracods, copepods, and euphausiids constituted 50.0, 21.2, 23.1, and 5.8%, respectively, of the identifiable crustacean prey.

## Discussion

*Halosauropsis macrochir* is an abundant deep-sea benthic fish species on the slopes and upper rise of the mid-Atlantic Ridge (MAR) [6], [7], and given the size of the habitat, the Mid-Atlantic Ridge (MAR) must constitute a significant proportion of the North Atlantic distributional range of the species. The size distributions suggest that the species completes its life cycle on the MAR, but whether there is connectivity with conspecifics on adjacent continental margins is unstudied.

In *H. macrochir* we observed no obvious depth trend in size composition. We used a single gear type and the abundance plots suggest that we sampled the entire depth range of the species. ‘Bigger-deeper’, and the opposite ‘smaller deeper’ trends, have been reported for deepwater fishes [15] and some such trends might reflect sampling artefacts, others were probably real. The halosaur showed neither of the two patterns. But size distributions from all depths were skewed towards the right and somewhat bimodal, suggesting that catchability increased with size. Small fish escaping under the groundrope and rockhopper gear of the trawl may underly this observation. In addition, small and large fish may be somewhat spatially segregated.

An interesting but unexplained uneven sex ratio was observed in the halosaur. The predominance of males contrasts with the dominance of females reported previously for the species [16] and also observed in other notacanthiform species [17], [18]. There may be sex-specific behavioural or small-scale distribution differences underlying this pattern. The trawl sampled flat soft-bottom areas only, i.e. not all other habitats on the ridge, and small differences in habitat preferences would not be detected.

Differences in growth between sexes appear common in deepwater species, as e.g. seen in *Coryphaenoides rupestris* where females are faster growing, have greater longevity and attain a greater asymptotic size than males [19]. In the halosaur we did not observe differences in length and weight at age between sexes. But minor differences may have been masked by the wide scatter in the observations, probably largely reflecting the relatively low precision in the age estimates.

Deepwater fishes are generally perceived as long-lived, e.g. [20]. This notion is rather strongly established but probably overly influenced by extremely long-lived species such as the orange roughy, *Hoplostethus atlanticus* [21], [22]. Life history strategies remain poorly understood for most deepwater species, and the diversity of strategies may be underestimated. For a fish of its size, the halosaur may be regarded as a K-species displaying a relatively long life-time and slow growth, but it is not an extremely long-lived species compared with many shallow-living fishes.

Efforts to develop age estimation techniques of deepwater species have increased in the last 35 years with many publications on otolith reading, analysis and their structures, e.g. [23]. The notion that the relative constancy of the deepwater production would result in continuous aseasonal somatic growth that would facilitate creation of annual growth zones in hard parts has been largely abandoned. Many deepwater species display patterns very similar to those accepted or assumed to represent annuli in shallow-living species, e.g. [24], [25], and for several species validation studies have confirmed that patterns may be regarded as annuli, e.g. [20], [21], [25], [26], [28]. Validation of our age readings was outside the scope of the study but is an obvious task for the future. Our results on age and growth of the halosaur presently rest on the assumption that the otolith growth zones we considered as annuli are in fact deposited on an annual frequency. The presumed annuli appeared similar to those of others species for which validation has been conducted (e.g. [20], [21], [25]–[30]).

The new diet data from the mid-Atlantic Ridge suffer from the common limitation that many prey items could only be assigned to higher taxonomical levels. The sample size is probably satisfactory for estimating proportions of main prey components, but not to characterise the diet in detail. Demersal and benthic deepwater fishes are generally opportunistic feeders. Four feeding strategies including opportunistic feeders or those focusing on a single prey were recognised by [31], [32], and ten trophic guilds were characterised by [33]. Most deep demersal species forage widely or are scavengers exploiting allochthonous organic input and may migrate to midwater areas to feed [15]. *Halosauropsis macrochir* on the mid-Atlantic Ridge would probably be regarded as a micronekton/epibenthos predator and possibly fits best within the Trophic Guild 3 of [33]. Although infauna was common in the diet on the mid-Atlantic Ridge, it was not as prominent as described previously on the American slope [8]. Based on their diet study, in which a high fraction of guts contained sediment, and behavioural observations from submersibles, they concluded that the halosaur was a benthic feeder. This would place it in Guild 4 of [33]. In our study we found infauna, epifauna, cephalopods and fish in the stomachs, and sediment was not common. Considering the entire predator size range, the halosaur would therefore rather be regarded as an active foraging euryphagous predator not fitting well within a single guild [33]. Whether some of the fish remains stemmed from carcasses and reflect scavenging cannot be excluded. Ontogenetic shifts in diet are poorly understood though a general pattern of increased prey size with predator size is common in most fishes, and this was also found in the halosaur, reflected mainly in the increasing fish fraction.

Our study of this common slope and rise species unfortunately did not comprise reproductive biology. Gravid females occurred year-round on the American slope [16]. Tasks for future studies are analyses of age and size at first maturation, fecundity, character and number of maturation cycles and spawning events, as well as reproductive behaviour. These are major remaining gaps added to the obvious challenge of validating the age estimates obtained from otoliths.
